# Continuous monitoring of diabetes with an integrated microneedle biosensing device through 3D printing

**DOI:** 10.1038/s41378-021-00302-w

**Published:** 2021-09-29

**Authors:** Yiqun Liu, Qi Yu, Xiaojin Luo, Li Yang, Yue Cui

**Affiliations:** 1 School of Materials Science and Engineering, Peking University, 100871 Beijing, P. R. China; 2 Renal Division, Peking University Institute of Nephrology, Peking University First Hospital, 100034 Beijing, P. R. China

**Keywords:** Electrical and electronic engineering, Materials science

## Abstract

Diabetes is a prevalent chronic metabolic disease with multiple clinical manifestations and complications, and it is among the leading causes of death. Painless and continuous monitoring of interstitial glucose is highly desirable for diabetes management. Here we unprecedentedly show continuous monitoring of diabetes with an integrated microneedle biosensing device. The device was manufactured with a 3D printing process, a microfabrication process, an electroplating process, and an enzyme immobilization step. The device was inserted into the dermis layer of mouse skin and showed accurate sensing performance for monitoring subcutaneous glucose levels in normal or diabetic mice. The detection results were highly correlated with those obtained from a commercial blood glucose meter. We anticipate that the study could open exciting avenues for monitoring and managing diabetes, alongside fundamental studies of subcutaneous electronic devices.

## Introduction

Diabetes is among the most prevalent and challenging diseases of the twenty-first century. Currently, approximately 10% of the world’s population has diabetes, and approximately 1.5 million people die directly from diabetes yearly^1,2^. In addition, long-term poor control of diabetes could induce several acute and chronic complications. For example, one-third to two-thirds of diabetic patients have cardiovascular and cerebrovascular diseases, renal diseases, eye diseases, or neuropathy^3,4^. Diabetic patients need to check their blood glucose levels frequently to take periodic medicines for glucose control^5,6^. However, the traditional fingerstick glucose test using commercial glucose meters could cause pain to patients, accompanied by possible infections and complications^7,8^. This test provides one glucose level at a time, and during diabetes management, a person needs to check their glucose levels several times a day. Thus, the traditional fingerstick glucose meter is quite inconvenient.

Continuous glucose monitoring (CGM) can enhance diabetes management; by timely learning the glucose level fluctuation to adjust the treatment plan, this technology can be used to reduce hospitalization, save medical costs, minimize ineffective drug utilization, and save lives^9,10^. Sweat, saliva, and tears are attractive body fluids for noninvasive diabetes monitoring, and various biosensing devices for measuring glucose in these fluids have been developed^11–13^. However, these devices may not be suitable for diabetes monitoring from a medical viewpoint since the correlations between the glucose levels in blood and the glucose levels in these fluids vary for different population samples^14–18^, thus yielding inaccurate diabetes monitoring. Interstitial fluid (ISF) is a small-volume compartment surrounded by cells that absorbs molecules from blood capillaries. Therefore, it contains rich physiological and metabolic information and provides molecular insight into a patient’s health state^19^. It has been demonstrated that the glucose levels in ISF excellently correlate with blood glucose levels^20,21^. There have been several commercial subcutaneous glucose sensors for continuous monitoring of diabetes, such as G6 (Dexcom, San Diego, USA)^22^, Freestyle Libre (Abbott, Chicago, USA)^23^, and Guardian (Medtronic, Northridge, USA)^24^. These sensors usually rely on the subcutaneous implantation of a needle-type biosensor with a length of several to ten millimeters to monitor glucose levels continuously for approximately 1 week^25,26^. However, these commercial sensors have several disadvantages, including skin pain, acupuncture phobia, and allergies^10,27^. Thus, a method for overcoming these drawbacks is highly desirable.

Microneedle systems have attracted great interest for various biomedical applications, such as drug delivery^28^ and diagnosis^29^. Through designing the structure of the microneedles, the microneedle systems could reach the dermis without reaching the pain points and cause little pain or no pain^30,31^. These systems could be safer for patients by lowering the possibility of infections^32,33^. Microneedles hold great promise for continuous and real-time monitoring of diabetes, and several research groups have tried to develop microneedle biosensors for diabetes monitoring^34–39^. However, these sensors had a limited glucose detection range—making the monitoring of the patients with a high glucose level inaccurate—and were not sensitive enough to reflect the real-time concentration of glucose or did not integrate the working, reference, and counter electrodes into one chip. Therefore, these devices cannot be used for diabetes monitoring.

In this study, we unprecedentedly show continuous monitoring of diabetes with an integrated microneedle biosensing device. The biosensing device is constructed with a simple procedure that enables it to monitor diabetes continuously. First, it is manufactured with a three-dimensional (3D) printing process to obtain the microneedle array. Next, a microfabrication process and an electroplating process are used to fabricate the electrochemical sensing electrodes on the microneedles, including a working electrode and a reference/counter electrode. Then glucose oxidase (GOD) is immobilized on the working electrode of the sensor. After the construction of the entire sensor, the microneedles are inserted into the dermis layer of mouse skin. In the presence of subcutaneous glucose, H_2_O_2_ is produced through an enzymatic reaction on the working electrode to generate a current signal response. During the fluctuation of the glucose levels in a normal or a diabetic mouse during a day, the subcutaneous glucose levels are monitored continuously and in real time. Furthermore, the signals from this device are compared with those from a commercial blood glucose meter to obtain its accuracy for diabetes monitoring.

## Results and discussion

### Sensing principles and materials characterization

**Fig. 1:**
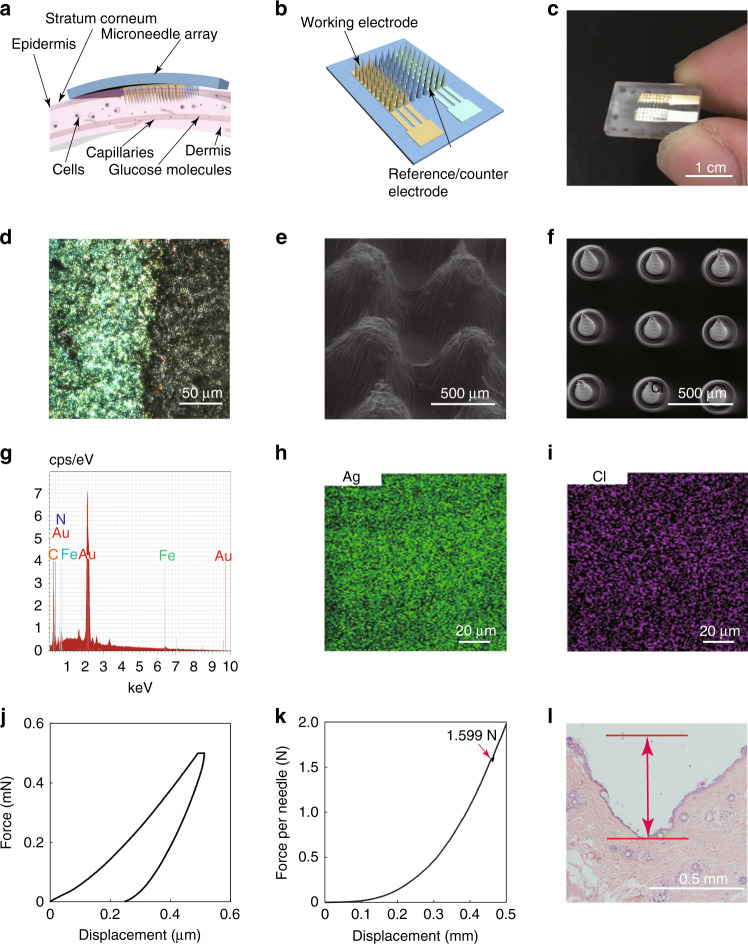
Overall scheme and materials characterization of the microneedle biosensing device. **a** A schematic illustration of the microneedle array inserted into the dermis of the skin and interstitial fluid. **b** A schematic illustration of the microneedle array. **c** A camera image of the microneedle-based electrochemical sensor. **d** An optical image of the Au electrode after the deposition of Prussian blue. **e** An SEM image of 3D printed cone-shaped microneedles with a base diameter of 400 µm and a height of 1.5 mm fabricated by using MoonRay (SprintRay Technology Ltd., China) and clear light-sensitive resin. **f** An SEM image of 3D printed cone-shaped microneedles with a base diameter of 200 µm and a height of 500 µm fabricated by using an S140 machine and biocompatible light-sensitive resin (BMF Precision Technology Ltd., China). **g** An EDS point analysis of the working electrode in the part of a microneedle. **h**, **i** EDS mapping of the Ag/AgCl electrode in part of a microneedle. **j** The load–displacement curve on a microneedle by an in situ nanomechanical test system. **k** Compression test on the microneedle array by a universal material testing machine. **l** An optical image of the pierced skin with staining after removing the microneedle array

Figure 1 shows the sensing principle and materials characterization of the microneedle biosensing device for diabetes. Figure 1a shows a schematic illustration of the microneedle biosensing device; the microneedle usually has a height of hundreds of micrometers, and it can penetrate the stratum corneum (10–20 μm) and epidermis (75–150 μm) layer to reach the dermis layer (1–4 mm)^40^. Due to their short height, users feel little or no pain and demonstrate little or no bleeding. ISF is surrounded by cells, and glucose molecules transmitted from the capillary endothelium diffuse there. The microneedle arrays ensure a sufficient contact area between the working electrode and skin ISF. By decreasing the device size, a stable sensor–skin interface is formed, and monitoring results are not affected significantly by the motions of users. Figure 1b reveals that the microneedle biosensor is constructed with a two-electrode configuration, including a Prussian blue-coated Au working electrode and an Ag/AgCl counter/reference electrode. Each electrode occupies a certain array of microneedles, and Au or Ag/AgCl is coated onto the microneedle surface.

Figure 1c shows a camera image of the sensing device with a two-electrode configuration. The microneedle array was fabricated by 3D printing with a clear resin. Au was coated onto the microneedles to form the working electrode in yellow, and Ag/AgCl was coated onto the microneedles to form the reference/counter electrode in a silver color. The working or reference/counter electrode area has a length of 5.2 mm and a width of 1.6 mm, with 27 microneedles in 9 rows and 3 columns. The size of the substrate was only 1 × 1.5 cm, and the microneedle array occupied a size of only 8.4 × 8.4 mm, which was very small for the human body. In practical applications, users could use a strong bandage or medical tape to fix it on skin that was not prone to wrinkles, such as the upper arm and abdomen, to avoid the effect of motion. Figure 1d shows an optical image of the Au electrode after the deposition of Prussian blue. The cone-shaped microneedle arrays can be printed with different sizes, as shown in the scanning electron microscopic images in Fig. 1e, f. The microneedle in Fig. 1e has a height of approximately 0.8 mm, a base diameter of 0.4 mm, and a space of 0.2 mm. The microneedle in Fig. 1f has a height of approximately 0.5 mm, a base diameter of 0.1 mm, and a space of 0.4 mm. Figure 1g shows the energy-dispersive spectrometry (EDS) point analysis of the Au electrode after the deposition of Prussian blue. Apart from the Au element, there are also obvious peaks of C, N, and Fe, which demonstrates that the Prussian blue (Fe_4_[Fe(CN)_6_]_3_) layer was successfully deposited on the Au electrode. Figure 1h, i show the EDS mapping of the Ag/AgCl electrode, which demonstrates that the Ag and Cl elements were evenly distributed on the sidewalls of the microneedles. The elastic modulus and hardness of the clear resin were measured to be 1.19 ± 0.07 and 0.079 ± 0.006 GPa using the nanomechanical test system (Fig. 1j), respectively. The fracture force of the microneedle was determined to be approximately 1.6 N per needle using universal material testing machining (Fig. 1k), which is sufficient for skin penetration without breaking from the theoretical analysis of the puncture force and fracture force for inserting microneedles into skin^41–43^. In the in vitro penetration test, the section of the puncture in mouse skin shows that the penetration depth was approximately 0.5 mm (Fig. 1l). These results demonstrate the successful insertion of the microneedle array into the skin. The difference between the height of the microneedle and the insertion depth may be attributed to the elastic deflection on the skin during microneedle insertion, resulting in part of the microneedle remaining outside the skin^44,45^.

### Electrode fabrication and sensing of H_2_O_2_

**Fig. 2:**
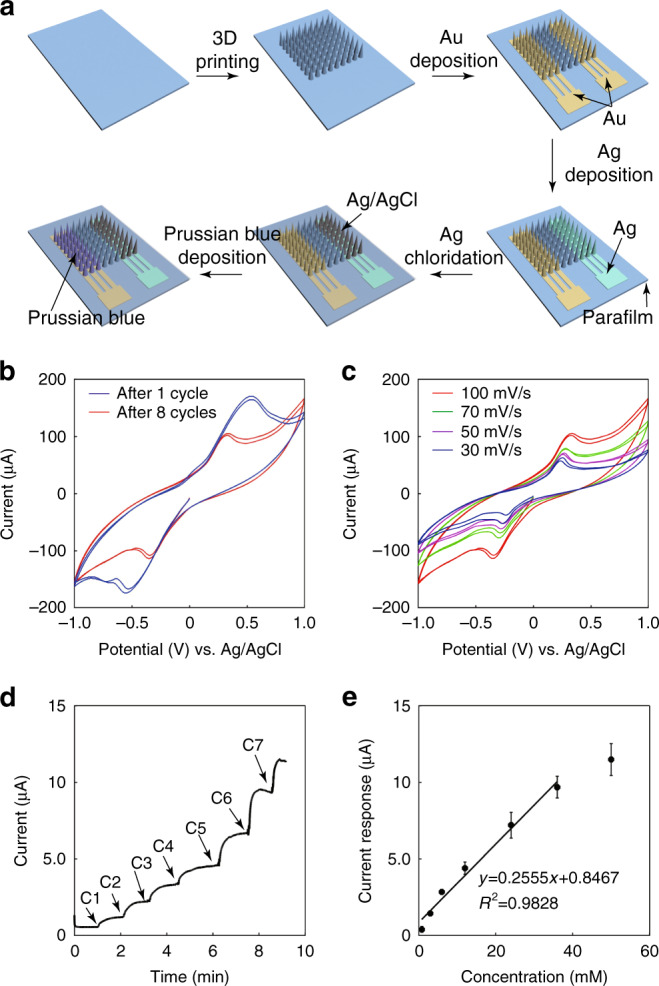
Fabrication process of the microneedle array sensor and its performance for detecting H_2_O_2_. **a** Schematic illustration of the preparation process for the biosensor. **b** Cyclic voltammograms of the sensor after the deposition of 1 and 8 cycles of Prussian blue. Solution: PBS containing 5 mM H_2_O_2_. Scanning rate: 100 mV/s. **c** Cyclic voltammograms of the sensor after the deposition of 8 cycles of Prussian blue. Solution: PBS containing 5 mM H_2_O_2_. Scanning rates: 30, 50, 70, and 100 mV/s. **d** Current versus time response curve upon the addition of H_2_O_2_ in PBS to the sensor. C1: 0.8 mM, C2: 2.2 mM, C3: 3.0 mM, C4: 6.0 mM, C5: 12 mM, and C6: 12 mM. **e** Calibration curve of the sensor for the detection of H_2_O_2_. Each error bar was from three sensors

Figure 2 shows the fabrication process of the integrated microneedle sensing device and its performance for detecting H_2_O_2_. Figure 2a shows that the microneedle arrays were manufactured by 3D printing. Next, Au evaporation was performed to obtain the working electrode and an adhesion layer of the reference/counter electrode. Then Ag was evaporated onto the adhesion layer to obtain a stable Ag electrode. Using chlorination, the Ag layer became an Ag/AgCl layer, acting as the reference/counter electrode of the sensor. Finally, an electroplating process was performed to obtain a Prussian blue layer on the Au working electrode. An integrated microneedle sensing device with a two-electrode configuration was then studied for electrode characterization and H_2_O_2_ sensing.

A layer of Prussian blue was used to extend the linear detection range of the biosensing device. Figure 2b shows the cyclic voltammograms (CVs) of the sensor after the deposition of Prussian blue with 1 cycle or 8 cycles. The solution was a phosphate buffer containing 5 mM H_2_O_2_. As shown in the figure, there was an obvious deviation in the oxidation peak, which demonstrated that Prussian blue was successfully deposited. Figure 2c shows the CVs of the microneedle sensing device for 5 mM H_2_O_2_ in phosphate buffer, with a voltage range from −1 to 1 V at different scan rates. As shown in the figure, there is a wide potential window for the oxidation or reduction of H_2_O_2_. Generally, when the potential value is larger, the oxidation or reduction rate of H_2_O_2_ is higher, and the current value is larger.

Figure 2d, e show the characterization of the integrated microneedle device for detecting H_2_O_2_—a product from glucose through enzymatic reactions with GOD. The successful detection of H_2_O_2_ indicates the possibility of constructing a glucose biosensor based on this electrode configuration. H_2_O_2_ was added onto the sensor, diffused to the surface of the working electrode, and then oxidized to produce a current signal. A working electrode potential of 0.6 V versus Ag/AgCl was used for detecting H_2_O_2_. As shown in Fig. 2d, by adding H_2_O_2_ onto the sensor surface, the current began to increase and reached a stable status in approximately 1 min, when the H_2_O_2_ concentration on the sensor became uniform and the time for quantifying the current was determined. A sequential addition of different concentrations of H_2_O_2_ to the sensor resulted in different current responses. Figure 2e illustrates the calibration curve between the current response and the concentration of H_2_O_2_. The current response increased linearly with increasing concentrations of H_2_O_2_, ranging from 0.8 to 36 mM. The slope of the calibration curve was 0.2555 ± 0.0277 μA/mM with a coefficient of 0.9828. The detection limit of this sensor for H_2_O_2_ in phosphate-buffered saline (PBS) was calculated to be 1.60 μM (signal-to-noise ratio of 3). These results demonstrate that the sensor can perform sensitive and rapid detection of H_2_O_2_.

### Selectivity and stability of the biosensing device

**Fig. 3:**
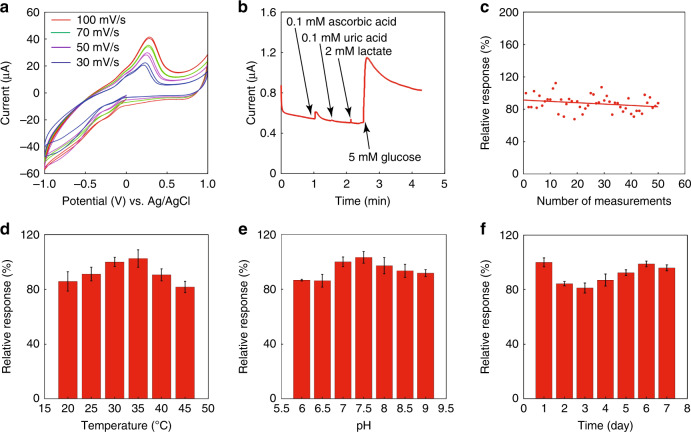
Selectivity and stability of the biosensing device in different environments. **a** Cyclic voltammograms of the biosensing device in PBS containing 5 mM glucose. Scanning rates: 30, 50, 70, and 100 mV/s. **b** Selectivity of the biosensing device to different interfering substances. **c** Measurement repeatability of the biosensing device. Glucose concentration: 5 mM in PBS. **d** Temperature stability of the biosensing device from 20 to 45 °C. Glucose concentration: 5 mM in PBS. **e** The pH stability of the biosensing device from pH 6 to 9. Glucose concentration: 5 mM in PBS. **f** The storage stability of the biosensing device over 7 days of storage. Glucose concentration: 5 mM in PBS (The sensor was measured three times under each condition, as shown in **d**–**f**)

Furthermore, GOD was immobilized on the working electrode to construct the entire biosensing device for glucose. Figure 3a shows the CV curve of the integrated microneedle biosensor for detecting 5 mM glucose in phosphate buffer solution, with a voltage range from −1 to 1 V. These curves show similar curve shapes with different scanning rates of 30, 50, 70, and 100 mV/s. At different potentials, there were different current values due to differences in the oxidation and reduction of H_2_O_2_ through the enzymatic reaction of glucose. These CV curves demonstrate that a potential range from 0.3 to 1 V could all generate a clear sensing signal, and a typical potential of 0.6 V could be used for further amperometric measurements.

The selectivity of the biosensing device was then studied. Device selectivity is crucial for monitoring diabetes accurately since the electrolytes and metabolites in the ISF may influence the sensor accuracy. The major interfering substances in the blood and ISF are ascorbic acid, uric acid, and lactate. Ascorbic acid and uric acid are electroactive substances. For both diabetic patients and normal people, ascorbic acid usually has a concentration of <0.1 mM in serum^46,47^, and uric acid usually has a concentration ranging from 0.1 to 0.4 mM in serum^48^. These concentrations are low enough and would not affect glucose sensing. Figure 3b shows the study of this device selectivity by comparing the signals from the interferences and glucose. As shown in the figure, although there are small current changes caused by the interferences, these signals are small enough compared to those generated by blood glucose or ISF glucose. These interferences would not cause a significant influence on the glucose sensing results. Therefore, the device should be able to perform accurate sensing of glucose.

The measurement repeatability of this sensor for detecting glucose was studied, as shown in Fig. 3c. The current response during the entire study tended to be stable and varied within 20%. The sensor could still maintain 87.6% of the initial response after repeated measurements over 50 times. The sensor stability was studied under different environments, including pH, temperature, and period (Fig. 3d–f). As shown in Fig. 3d, the sensor is stable at temperatures from 20 to 45 °C, with the lowest relative response of 81.7% at 45 °C. The sensor was studied at different pH values (Fig. 3e). The sensor shows a stable performance from pH 6 to pH 9, and the lowest relative response is 86.2% at pH 6. The sensor was studied for 7 days (Fig. 3f). The sensor was stable for over a week, and on the seventh day, the relative response was 93.9%. For the study of pH, temperature, and storage stabilities, the range of the noise is from 0.61 to 7.05%. For measuring 5 mM glucose, the calculated noise concentration ranged from 0.04 to 0.43 mM. In terms of the measured glucose concentration of 5 mM, it is small enough, and the ability to quantify ISF glucose is sufficient. Timely calibration may eliminate the small errors caused by changes in the environment and the activity loss of GOD. These results demonstrate that the biosensing device has excellent stability.

### In vitro sensing of glucose

**Fig. 4:**
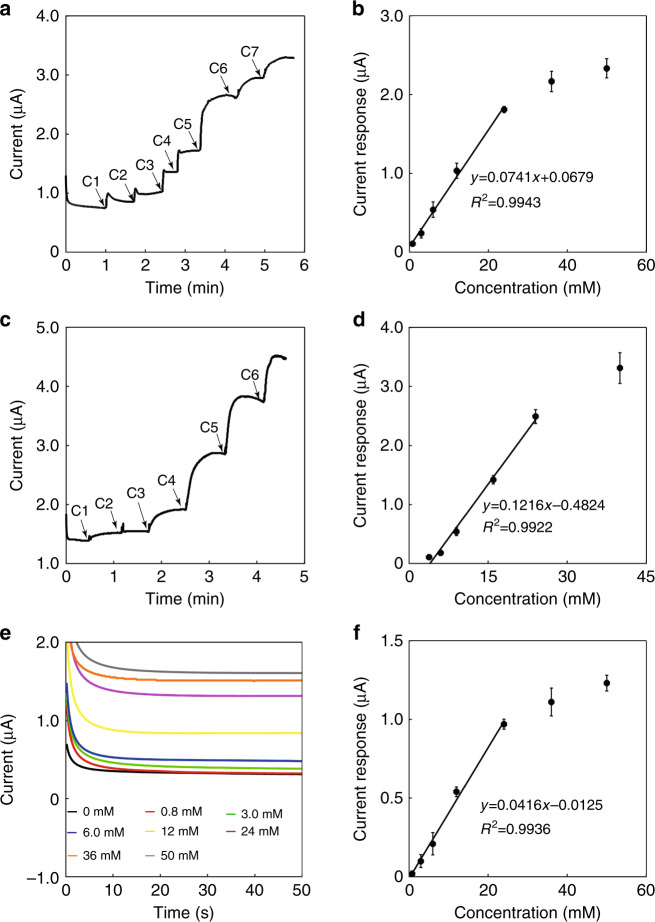
In vitro sensing of glucose in different solutions with the biosensing device. **a** Current versus time curve upon the addition of glucose in PBS. (C1: 0.8 mM, C2: 2.2 mM, C3: 3.0 mM, C4: 6.0 mM, C5: 12 mM, C6: 12 mM, C7: 14 mM). **b** Calibration curve for the detection of glucose in PBS. **c** Current versus time curve upon the addition of glucose to goat plasma (C1: 0.8 mM, C2: 2.2 mM, C3: 3.0 mM, C4: 7.0 mM, C5: 8.0 mM, C6: 16 mM). **d** Calibration curve for the detection of glucose in goat plasma. **e** Current versus time curve from simulated interstitial fluids with different glucose concentrations. **f** Calibration curve for the detection of glucose in simulated interstitial fluids. Each error bar was from three sensors

The biosensing device was further characterized for the in vitro detection of glucose. Phosphate buffer solution is a simple solution environment, and it was first studied for sensing glucose. Figure 4a shows the sequential addition of different glucose concentrations into a phosphate buffer solution, and the current signal increases as the glucose concentration increases. The point to quantify the current was approximately 40 s after each addition of glucose. Figure 4b shows the calibration curve of the microneedle biosensing device for detecting glucose in the buffer solution. A linear detection range was clearly obtained from 0.8 to 24 mM with a sensitivity of 0.0741 ± 0.0004 μA/mM. The saturation effect was obvious at higher glucose concentrations due to the limited catalytic ability of GOD. The detection limit of this sensor for glucose in PBS was calculated to be 15.4 μM (signal-to-noise ratio of 3).

Next, the sensing behavior of the microneedle biosensing device was studied in goat plasma with glucose concentrations ranging from 3 to 40 mM (Fig. 4c, d). The point to quantify the current was approximately 40 s after each addition of glucose, as shown in Fig. 4c. The original glucose concentration of the plasma was approximately 3 mM, measured by a commercial blood glucose meter 3 times. Different amounts of glucose were added to the plasma. The studied glucose concentration range corresponded to a typical range of blood glucose for normal and diabetic patients. The sensor could detect glucose accurately with a linear concentration range from 3 to 24 mM and a linear slope of 0.1216 ± 0.0044 μA/mM. The detection limit of this sensor for glucose in goat plasma was calculated to be 48.1 μM (signal-to-noise ratio of 3). The device also demonstrated great performance in goat plasma with a higher initial glucose level of 8 mM (see Supplementary Material Fig. S1).

Furthermore, we verified the sensing performance in a simulated ISF. The simulated ISF behaves like a hydrogel, making it unable to perform the sequential addition of glucose into the simulated ISF. Thus, simulated ISFs with different glucose concentrations were prepared. Figure 4e shows the current responses of the biosensing device to different concentrations of glucose in the simulated ISFs. The time for quantifying the current was 50 s after placing the sensor in the simulated ISF. It shows that different glucose concentrations result in different current values, and a higher concentration has a larger current value. Figure 4f shows the calibration curve for detecting glucose. The sensor shows a linear detection range from 0.8 to 24 mM with a slope of 0.0416 ± 0.0028 μA/mM. The detection limit of the sensor for glucose in simulated ISF was calculated to be 8.65 μM (signal-to-noise ratio of 3). These results indicate the possibility that the integrated microneedle biosensing device could be used for the accurate and continuous monitoring of subcutaneous glucose.

### In vivo monitoring of subcutaneous glucose in mice

The safety of microneedles has been the focus of concern for researchers. To evaluate the in vivo biocompatibility of this device, an in vivo dermal irritation experiment was conducted (see Supplementary Material Fig. S2). The result indicates that the clear resin and electrode are relatively not biocompatible and harmful to skin. However, the biosafety of this device could be further enhanced by covering the microneedle array with a biocompatible membrane, such as Nafion.

**Fig. 5:**
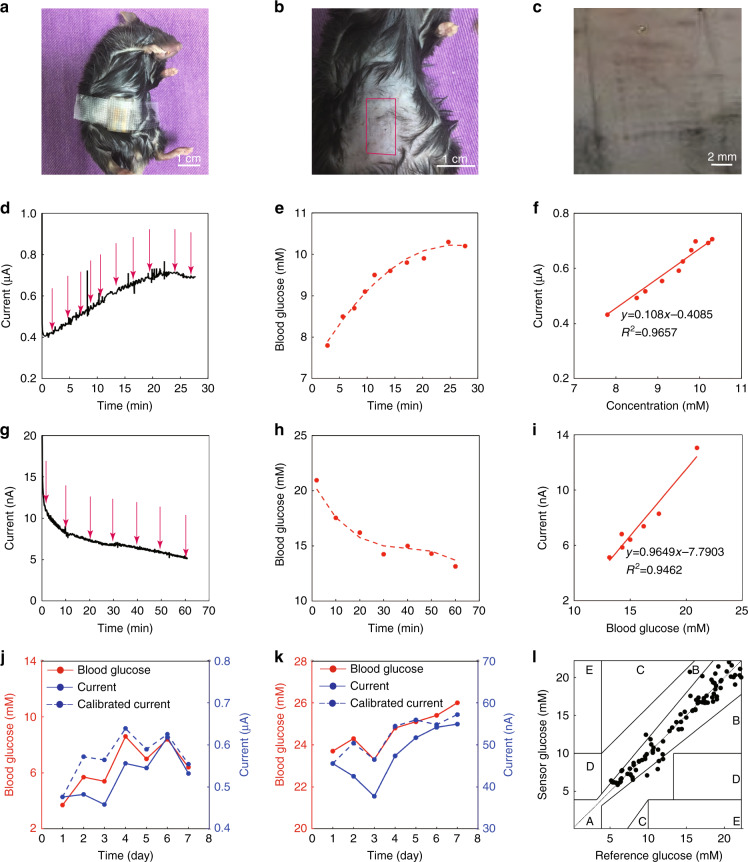
In vivo monitoring of subcutaneous glucose in mice with the biosensing device. **a** A camera image of the microneedle array applied to a mouse. **b** A camera image of a mouse’s skin on the abdomen after the application of the microneedle array biosensor. **c** A magnified image of the needle pores left on the mouse’s skin after the application of the microneedle array biosensor. **d** Current versus time curve of the biosensing device for detecting subcutaneous glucose in a normal mouse after being injected with glucose. Blood glucose was measured at the time indicated by the arrow by using a commercial blood glucose meter. **e** Blood glucose versus time curve for a normal mouse after being injected with glucose, measured by a commercial blood glucose meter. **f** Correlation between the subcutaneous glucose levels measured by the microneedle biosensing device and the blood glucose levels measured by a commercial glucose meter for a normal mouse after being injected with glucose. **g** Current versus time curve of the biosensing device for detecting subcutaneous glucose in a diabetic mouse after being injected with insulin. Blood glucose was measured at the time indicated by the arrow by using a commercial blood glucose meter. **h** Blood glucose versus time curve for a diabetic mouse after being injected with insulin, measured by a commercial blood glucose meter. **i** Correlation between the subcutaneous glucose levels measured by the microneedle biosensing device and the blood glucose levels measured by a commercial glucose meter for a diabetic mouse after being injected with insulin. **j** Seven-day monitoring of the subcutaneous glucose levels in a normal mouse by the microneedle biosensing device (blue), in comparison with a commercial blood glucose meter for blood glucose (red). **k** Seven-day monitoring of the subcutaneous glucose levels in a diabetic mouse by the microneedle biosensing device (blue), in comparison with a commercial blood glucose meter for blood glucose. **l** Clark error grid for the microneedle biosensing device. The *x*-axis represents the reference blood glucose values measured by a commercial blood glucose meter, and the *y*-axis displays the glucose values measured by the microneedle biosensing device (the data were from eight normal and eight diabetic mice)

Figure 5 shows the integrated microneedle biosensing device for the continuous in vivo monitoring of subcutaneous glucose levels in normal and diabetic mice. Figure 5a shows the successful application of the microneedle arrays onto a mouse with medical tape being used to fix the device. Figure 5b, c shows camera images of a mouse’s abdomen skin after the application of the microneedle arrays for 5 min. Structural changes on the skin after inserting and removing the microneedle arrays were observed. It clearly exhibits the patterns of the pores paralleling the shapes and patterns of the microneedle arrays.

Figure 5d–f show the sensing performances of the microneedle biosensing device for the real-time and continuous monitoring of subcutaneous glucose in a normal mouse injected with a glucose solution (food for a mouse). During diabetes management, any diabetic patient consumes meals every day, and after the intake of food, the blood sugar of the diabetic patient increases. This is to mimic the situation of eating food. Figure 5d shows the current trend for a normal mouse after being injected with glucose, which clearly shows an increase in the current. Concurrently, the blood glucose levels of a normal mouse increased after the injection of glucose (Fig. 5e). The blood glucose concentrations were measured using a commercial blood glucose meter. The sampling intervals (approximately 200 s) in Fig. 5d, e were synchronized. For comparison, a linear curve was plotted between the sensing results from the microneedle biosensing device and the detection results from the commercial blood glucose meter. An excellent linear relationship was observed, with a slope of 0.108 μA/mM and an *R*^2^ of 0.9657 (Fig. 5f). The results demonstrate that, during the intake of sugar, the detection results of the subcutaneous glucose concentrations measured by the microneedle biosensing device correlate excellently with those from the blood glucose concentrations measured by a commercial glucose meter.

Figure 5g–i shows the performance of the microneedle biosensing device for continuous monitoring of subcutaneous glucose in a diabetic mouse after being injected with insulin. During diabetes management, insulin is frequently used. This is to mimic the situation of insulin treatment for diabetic patients. Figure 5g shows that the current decreased continuously after the injection of insulin. In addition, the blood glucose levels of a diabetic mouse decreased concurrently (Fig. 5h). This phenomenon is within the expectation since insulin would decrease the glucose concentration for diabetic patients. A linear relationship was observed between the currents from the microneedle biosensing device (Fig. 5g) and the blood glucose levels from a commercial glucose meter (Fig. 5h), with a slope of 0.9649 nA/mM and an *R*^2^ of 0.9462. The results demonstrate that, during diabetes management with insulin, the detection results of the subcutaneous glucose concentrations from the microneedle biosensing device excellently correlate with those from the commercial glucose meter. The decrease in the biosensor sensitivity in diabetic mice may be due to the higher resistance of diabetic mouse abdominal skin (>100 MΩ/cm) than that of normal mice (13.1 ± 2.9 MΩ/cm, these data are from three mice measured using a digital multimeter). Therefore, the calibration procedure is necessary for further practical applications.

Figure 5j, k show the results of the device for 7-day glucose monitoring in normal and diabetic mice. The current value on each day was recalibrated according to its relative response over 7 days (Fig. 3f) and shows that the interstitial glucose level determined by using the microneedle biosensing device has a strong correlation with blood glucose over 7 days with a high correlation coefficient of 0.9032 for the normal mouse and 0.9239 for the diabetic mouse.

The conventional Clark error grid was used to analyze the comparison between the reference blood glucose value and the glucose value measured by this device (the data came from eight normal and eight diabetic mice). A 45-degree line was obtained for all 118 points, and they were located in clinically acceptable error zones A (meaning no effect on clinical action) and B (representing small altered clinical action or no significant effect on clinical outcome). The mean absolute relative difference value of the reference blood glucose value and the device sensing value was calculated to be 7.20%, which shows the excellent accuracy and safety of the sensor in monitoring glucose levels. Although there is a time lag of approximately 5 min between the changes in interstitial glucose concentration and blood, it could be calibrated by an algorithm in actual applications^49,50^.

These results demonstrate that the microneedle biosensing device can monitor subcutaneous glucose continuously in both situations, including the intake of food and the injection with insulin. The sensor shows accurate monitoring for either situation, and the detection results from the microneedle biosensing device excellently correlate with those from a commercial blood glucose meter. These two situations are the main causes of fluctuations in glucose levels in the body. The results demonstrate that the sensor can potentially be worn by a patient continuously from several hours to several days during diabetes management.

## Conclusion

We have unprecedentedly demonstrated an integrated microneedle biosensing device for the continuous monitoring of subcutaneous glucose in normal and diabetic mice. The biosensing device showed reliable and stable detection of glucose in buffer solution, plasma, and simulated ISF, and the microfabrication and electrochemical plating steps enabled the sensor to detect glucose linearly and sensitively with a broad detection range. Furthermore, the sensor was inserted into the dermis layer of mouse skin, and the sensor showed continuous and real-time monitoring of the subcutaneous glucose levels accurately under the intake of food or insulin injection. Although these results are promising, further studies are needed to help elucidate the roles of the microneedle structure and skin thickness for the continuous in vivo monitoring of diabetes.

## Materials and methods

### Fabrication of the microneedle array

A digital light processing machine (from MoonRay, SprintRay Technology Ltd. or S140, BMF Precision Technology Ltd., China) was employed to fabricate the device. The original 3D model of a microneedle has a conical shape with a base diameter of 400 μm and a height of 1.5 mm. A 9 × 9 microneedle array was designed and printed, and the center-to-center distance of two adjacent microneedles was 600 μm. The sharp tip structure enabled the microneedle to stick easily into the skin. The substrate was made of the same materials as the microneedles, having a size of 2 mm in thickness, 1 cm in width, and 1.5 cm in length.

The resolution of the 3D-printing machine greatly influences the height and structure of microneedles. The x-axis and *y*-axis resolutions of MoonRay (SprintRay Technology Ltd., China) were 100 μm, the layer height was 20 μm, and the materials used for printing were a clear light-sensitive resin (SprintRay Technology Ltd., China). The S140 machine (BMF Precision Technology Ltd., China) had a higher resolution (the *x*-axis and *y*-axis resolution could be 10 μm, and the layer height was 10 μm), and the materials used for printing were a biocompatible light-sensitive resin (BMF Precision Technology Ltd., China).

### Sensing electrode preparation

The Au and Ag/AgCl electrodes were separated by three columns of microneedles in the middle, and each occupied three microneedle columns. A shadow mask was designed and fabricated in stainless steel by laser irradiation. The microneedle array was first cleaned with ultraviolet (UV) ozone (Ossila Co., UK) for 10 min. To fabricate the Au electrode, a 20-nm-thick adhesive Ti layer and a 200-nm-thick Au layer were sequentially deposited on the needle substrate using magnetron sputtering with a direct-current sputtering model. To fabricate the Ag/AgCl electrode, a 200-nm-thick Ag layer was further deposited on the reference/counter electrode area. Then a parafilm barrier (~100 μm thick) was employed, and the microneedles punctured the parafilm and immersed into the solutions for further experiments.

Next, the two-electrode system was first immersed in 0.5 M H_2_SO_4_ for a CV measurement (A CHI660e, from CH Instruments, China), and it was performed at a rate of 1 V/s with a voltage ranging from 0.2 to 1.2 V for 10 cycles. CV scanning in sulfuric acid could remove impurities from the electrode surface and activate the electrode. Then a 50 mM ferric chloride (FeCl_3_) solution was incubated with the Ag surface for 30 s to chlorinate the Ag layer and grow a AgCl layer. Furthermore, a Prussian blue (including 2.5 mM FeCl_3_, 100 mM KCl, 2.5 mM K_3_Fe(CN)_6_, and 100 mM HCl) mediator layer was deposited onto the Au working electrode by cyclic voltammetry from −0.15 to 0.3 V (vs. Ag/AgCl) for 8 cycles at a rate of 20 mV/s. A thinner Prussian blue layer could provide a wider measurement range, which was essential for detecting a high glucose level in the ISF. Finally, the electrodes were immersed in a 0.1 M KCl/HCl solution with cyclic voltammetry from −0.2 to 0.5 V (vs. Ag/AgCl) for 4 cycles at 50 mV/s to stabilize the Prussian blue electrode.

### Enzyme functionalization

The fabricated sensor was first treated with UV ozone for 10 min to obtain a hydrophilic surface to improve its contact with the enzyme. A diluted glutaraldehyde (2%) was mixed with GOD (Toyobo Ltd., Toyobo) solution (50 U/μl) at a volume ratio of 1:1, and then 20 μl of the mixture was deposited on the Au working electrode. These sensors were finally dried and stored in a refrigerator at 4 °C.

### Electrical sensing measurement

The sensing measurement was performed at room temperature (24 °C). A potentiostat (A CHI660e, from CH Instruments, China) was used with a constant voltage of 0.6 V at the Au working electrode (versus the Ag/AgCl electrode) to measure the currents of the device under different circumstances. The working electrode lead from the potentiostat was connected to the Au working electrode of the sensor. The reference and counter electrode leads from the potentiostat were connected to the Ag/AgCl electrode of the sensor. The currents of the device were recorded to plot the response and calibration curves. Commercial glucose meters (Sinocare Inc., China) were used to compare the detection results.

### Simulated sensing environment

The sensing performances were first studied under different simulated environments, including a pure buffer solution, simulated ISF, and a plasma environment. In a pure buffer environment (50 mM phosphate buffer, pH 7.0), 50 μl of the buffer was first dropped on the sensor, and 2 μl of different concentrations of glucose or H_2_O_2_ were added incrementally onto the working electrode. Under the simulated ISF environment, the sensor was immersed in simulated ISFs with different glucose concentrations. The simulated ISF was prepared by mixing alginic acid viscous solution with 1.5% w/v in 0.1 M KCl for 4 h at 45 °C, followed by titration with 0.2 M calcium chloride to form a hydrogel-like solution. Under the plasma environment, 50 μl of goat plasma (Senber Biotechnology Co., China) was dropped onto the sensor, and 2 μl of different concentrations of glucose were added incrementally onto the working electrode.

### Mechanical property test

An in situ nanomechanical test system (TI-900 TriboIndenter, Hysitron, Ltd., USA) was used to extract the elastic modulus and hardness of the microneedle. The load and depth of penetration were recorded to plot the load–displacement curve during the testing process.

A universal material testing machine (MTS SYSTEMS (China), Co., Ltd., China) was used to study the mechanical strength of microneedles under compression testing. The microneedle array was placed on the bottom circular plate workbench. The initial gauge was set to 2.00 mm between the tips of the microneedles and the top circular plate workbench. The speed of the top workbench moving toward the microneedles was 0.1 mm/min. The load and displacement were recorded every 0.01 s to obtain the load–displacement curve.

### In vitro penetration ability test

The abdominal skin of the mouse was used as the model to investigate whether the microneedle array could be inserted into the skin. The shaved skin was placed on a horizontal plate, and a microneedle array was applied to the skin for approximately 1 min. Then the pierced skin was stained with hematoxylin and eosin after removing the microneedle array.

### Mouse experiments

Male C57BL/6J nondiabetic mice and C57BL/6J db/db diabetic mice (20–22 g) were purchased from Jiangsu GemPharmatech Co., Ltd. (Jiangsu, China) [License No. SCXK (Su) 2018–0008]. Specific pathogen-free conditions were provided to all mice, and all procedures of the animal experiments were approved by the Institutional Animal Care and Use Committee of Peking University First Hospital (Approval Number: J201957).

Mice were maintained in individually ventilated cages and had access to standard laboratory food and water during free time. Before conducting the experiments, all mice were fasted for 8 h and were anesthetized appropriately using the anesthetic agent pentobarbital. Then glucose was intraperitoneally injected into the nondiabetic mice (10% glucose at 0.1 ml/10 g), and the diabetic mice received insulin (0.5 U/ml at 0.054 ml/10 g). Before applying the biosensor onto the mouse, the insertion site of the mouse’s skin was shaved and cleaned. Then the microneedle arrays of the sensor were inserted into the mouse’s skin, and a period of 30 min was needed for the sensor to warm up and stabilize. To measure the changes in the blood glucose levels in a mouse after injecting glucose or insulin, blood samples were collected from the tail vein of each mouse at different times and measured using a commercial glucose meter.

## Supplementary information

Supplementary Material
