# Supplementary material for: Continuous monitoring of diabetes with an integrated microneedle biosensing device through 3D printing
Source: Microsyst Nanoeng. 2021 Sep 29;7:75. doi: 10.1038/s41378-021-00302-w (PMC8481261; doi:10.1038/s41378-021-00302-w)
Supplement: Supplementary file 1 — Supplementary Material [file 41378_2021_302_MOESM1_ESM.docx]

Supplementary Material for

Continuous Monitoring of Diabetes with an Integrated Microneedle Biosensing Device through 3D Printing

Yiqun Liu^1^, Qi Yu^2^, Xiaojin Luo^1^, Li Yang^2^*, Yue Cui^1^*

^1^School of Materials Science and Engineering, Peking University, Beijing 100871, P.R. China

^2^Renal Division, Peking University Institute of Nephrology, Peking University First Hospital, Beijing 100034, P.R. China

E-mail: [ycui@pku.edu.cn](mailto:ycui@pku.edu.cn), [li.yang@bjmu.edu.cn](mailto:li.yang@bjmu.edu.cn)

**Theoretical analysis of the puncture force and fracture force for inserting microneedles into the skin.**

The microneedle may fail under five ultimate loads during the process of piercing the skin: the compression force, the buckling force, the free bending force, the constrained bending force and the maximum shear force^1^.

The maximum compressive force that the microneedle can withstand without breaking is^1^:

$$F_{maximum compressive force}=\sigma_{y}A$$

Where the $\sigma_{y}$ is the fracture strength of materials to fabricate microneedle and A is the cross-sectional area of the microneedle.

The maximum buckling force that the microneedle can withstand without breaking is^1^:

$$F_{maximum buckling force}=\frac{\pi^{2}EI}{4L^{2}}$$

Where the E is the Young’s Modulus of materials to fabricate microneedle, I is the moment of inertia and L is the length of the microneedle. This formula is valid only for “long” MNs with a large slenderness ratio. For microneedles with a small slenderness ratio, the Johnson formula should be used^2^.

The maximum free bending force that the microneedle can withstand without breaking is^1^:

$$F_{maximum free bending force}=\frac{\sigma_{y}I}{cL}$$

Where the c is the distance of the neutral axis to the outermost edge of the microneedle.

The maximum constrained bending force that the microneedle can withstand without breaking is^1^:

$$F_{maximum constrained bending force}=\frac{2\sigma_{y}I}{cL}$$

The maximum shear force that the microneedle can withstand without breaking is^1^:

$$F_{maximum shear force}=\frac{\sigma_{y}A}{2}$$

Among these forces mentioned above, the essential one is the maximum buckling force, due to its smallest value. Only when the maximum buckling force is much higher that the maximum force for insertion, The microneedle can resist all other forces.

The maximum force for insert the microneedle into the skin depends on the tip diameter of the microneedle, the initial force F_0_, the skin’s puncture toughness G_P_, and characteristic insertion length λ. It can be calculated as follows^3^:

$$F_{maximum insertion force}=F_{0}+\frac{G_{P}A}{\lambda}$$

This force falls drastically once the skin is punctured^4,5^. After the skin is pierced, the only force that acts on the microneedle is the frictional force due to tissue clamping the needle, which remains constant at constant needle velocity. This G_P_ depends on the skin’s biomechanical properties that different in healthy and non-healthy skin, and ages^6^.

The critical fracture force for the microneedle could be considered as^3^:

$$F_{critical fracture force}=\pi Dsin\alpha\omega_{t}\sigma_{y}$$

where D is the diameter of the needle tip, α is wall angle of the microneedle, $\omega_{t}$is the wall thickness of the microneedle.

The following relationship need to be considered for the safe insertion of microneedles:

$$F_{maximum insertion force}<F_{maximum buckling force}\leq F_{critical fracture force}$$

The fracture force of the microneedle is determined to be ~1.6 N per needle by using the universal materials testing machine (Fig. 4j), which is sufficient for skin penetration without breaking^1,3,7^.


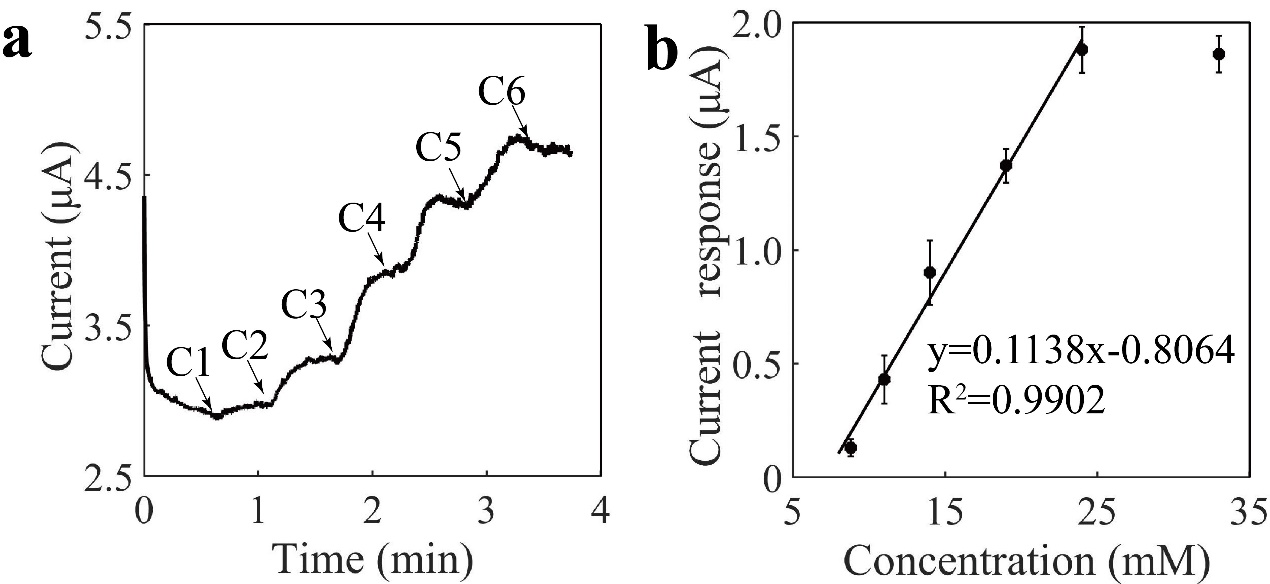


Fig. S1 In-vitro sensing of glucose in goat plasma with higher initial glucose levels. a Current-verses-time curve upon the additions of glucose in goat plasma with higher initial glucose levels (C1: 0.8 mM, C2: 2.2 mM, C3: 3.0 mM, C4: 7.0 mM, C5: 8.0 mM, C6: 8.0 mM). b Calibration curve for the detection of glucose in goat plasma. Each error bar was from three devices.

**In-vivo biocompatibility test**


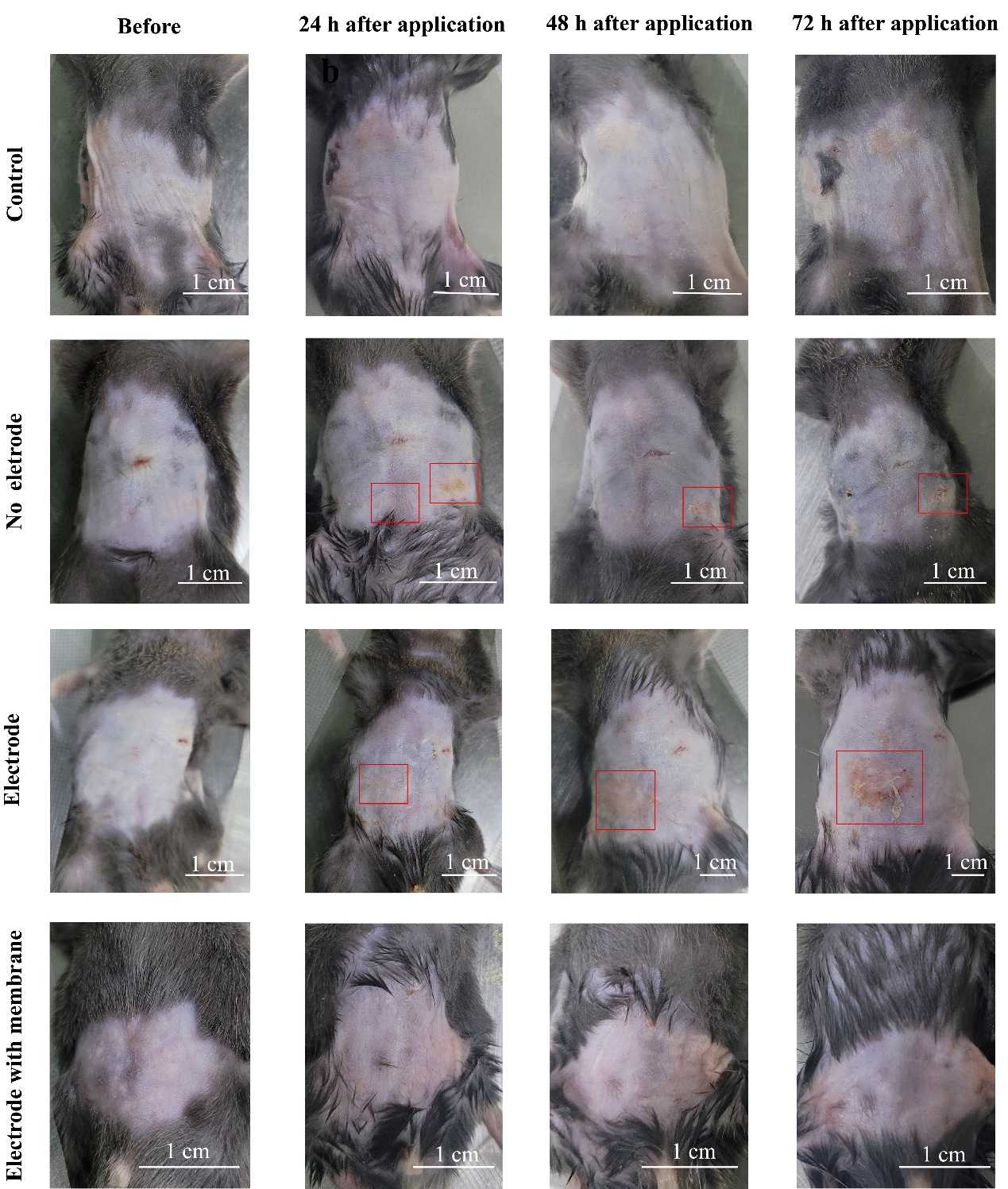


Fig. S2 Camera images of the rat skin after the dermal irritation test before and removal of the device.

The dermal irritation experiment was conducted to evaluate the in-vivo biocompatibility of the device. The fur of each mouse from the dorsal area was removed, 24 h prior to the application of the device. Just before the application, each mouse was carefully checked to determine that there were no abnormalities on the skin. Then the device was directly applied on the shaved intact skin and fixed by using the medical tape. Four mice were used to investigate the in-vivo biocompatibility of the clear resin, the electrode with enzyme immobilization, the electrode covered with the Nafion membrane. The control mouse was only applied with medical tape, the first mouse was applied with the microneedle array without electrode, the second mouse was applied with microneedle array with electrode immobilized with enzymes, and the third mouse was applied with the microneedle array covered with the Nafion membrane. The skin sensitization reactions, including erythema and edema, were observed and recorded at 24 h, 48 h and 72 h after application and compared with the control mouse.

The results of dermal irritation experiment are showed in Fig. S3. Compared to the control mouse (without device application) and excluding the abnormalities caused by the medical tape, there were erythema and oedema observed after application of the device for 24 h, 48 h and 72 h in the no electrode and electrode mouse. However, there is no obvious erythema, oedema and any inflammation observed on the mouse skin applied with the microneedle array covered with a Nafion membrane,

**References**

1 Aggarwal, P. & Johnston, C. R. Geometrical effects in mechanical characterizing of microneedle for biomedical applications. *Sens. Actuat. B-Chem.* **102**, 226-234, doi:10.1016/j.snb.2004.04.024 (2004).

2 Park, J. H. & Prausnitz, M. R. Analysis of the Mechanical Failure of Polymer Microneedles by Axial Force. *J. Korean Phys. Soc.* **56**, 1223-1227, doi:10.3938/jkps.56.1223 (2010).

3 Davis, S. P., Landis, B. J., Adams, Z. H., Allen, M. G. & Prausnitz, M. R. Insertion of microneedles into skin: measurement and prediction of insertion force and needle fracture force. *J. Biomech.* **37**, 1155-1163, doi:10.1016/j.jbiomech.2003.12.010 (2004).

4 Frick, T. B., Marucci, D. D., Cartmill, J. A., Martin, C. J. & Walsh, W. R. Resistance forces acting on suture needles. *J. Biomech.* **34**, 1335-1340, doi:10.1016/s0021-9290(01)00099-9 (2001).

5 Brett, P. N., Fraser, C. A., Hennigan, M., Griffiths, M. V. & Kamel, Y. Automatic surgical tools for penetrating flexible tissues. *IEEE Eng. Med. Biol. Mag.* **14**, 264-270, doi:10.1109/51.391778 (1995).

6 Pawlaczyk, M., Lelonkiewicz, M. & Wieczorowski, M. Age-dependent biomechanical properties of the skin. *Postep. Derm. Alergol.* **30**, 302-306, doi:10.5114/pdia.2013.38359 (2013).

7 Zhang, Y., Jiang, G. H., Yu, W. J., Liu, D. P. & Xu, B. Microneedles fabricated from alginate and maltose for transdermal delivery of insulin on diabetic rats. *Mater. Sci. Eng. C-Mater. Biol. Appl.* **85**, 18-26, doi:10.1016/j.msec.2017.12.006 (2018).
